# The efficacy of pivmecillinam: 3 days or 5 days *t.i.d* against community acquired uncomplicated lower urinary tract infections – a randomized, double-blinded, placebo-controlled clinical trial study protocol

**DOI:** 10.1186/s12879-016-2022-0

**Published:** 2016-12-01

**Authors:** Filip Jansåker, Niels Frimodt-Møller, Lars Bjerrum, Jenny Dahl Knudsen

**Affiliations:** 1 The Department of Clinical Microbiology, Copenhagen University Hospital, Hvidovre Hospital, Kettegård Allé 30, 2650 Hvidovre, Denmark; 2 The Department of Clinical Microbiology, Copenhagen University Hospital, Rigshospitalet, Blegdamsvej 9, 2100 Copenhagen, Denmark; 3 Section of General Practice and Research Unit of General Practice, Department of Public Health, University of Copenhagen, Oester Farimagsgade 5, 1014 Copehangen K, Denmark

**Keywords:** Cystitis, UTI, Pivmecillinam, General practice, Study protocol

## Abstract

**Background:**

Uncomplicated lower urinary tract infections (LUTI) are very common, and presumably around 200,000 female patients are treated for this annually in Denmark. The current Danish national clinical practice guidelines recommend pivmecillinam as a first-line drug (i.e.*,* 400 mg t.i.d. for 3 days). Pivmecillinam is also one of the first-line drugs recommended in the international guidelines for LUTIs (i.e.*,* 400 mg b.i.d. for 5 days). The international recommended duration is based on evidence saying that a 7-day regimen is better than a 3-day regimen. However, no data says that a 5-day regimen is superior to a 3-day regimen. With this study we aim to identify and to compare the efficacy of pivmecillinam 400 mg t.i.d in a 3-day respectively 5-day regimen, against community acquired uncomplicated LUTI, i.e.*,* in women at the age of 18–70 year old.

**Method/design:**

The general practitioner will at consultation give a suitable patient the opportunity to participate in the study. If the patient will give her consent, a double-blinded kit (i.e.*,* the antibiotic with/without placebo, questionnaires and self-urinary samples) will be given to the patient. We aim for 161 evaluable patients in each arm.

**Discussion:**

Pivmecillinam is an excellent choice against urinary tract infections and we believe this study will fill in the gaps and strengthen the evidence on the treatment against one of the most common infections in our society. Thus, aiming to provide a more rational and ecological beneficial antimicrobial therapy.

**Trial registration:**

EudraCTno.: 2014-001321-32.

## Background

Uncomplicated lower urinary tract infections (LUTIs) are very common (i.e. 10% of all women has it each year and 60% of all women experience at least one episode during their lifetime) [1], and we presume there are about 200,000 patients treated for this in Denmark annually. Most patients are otherwise healthy women, and these infections are often the main reason of exposure to antibiotics for these patients [2]. Uncomplicated LUTIs are primarily community acquired and diagnosed as well as treated in primary care. The most common pathogen is *Escherichia coli* [2, 3], and diagnosis can be made clinical with 90% positive predictive value (i.e. new onset dysuria, pollakisuria and urgency, together with absence of vaginal discharge or pain) [4].

In Denmark, the current clinical practice recommendations is pivmecillinam (i.e.*,* 400 mg *t.i.d* for 3 days) as the first line drug against uncomplicated LUTIs [5]. Pivmecillinam is a prodrug of mecillinam. It is an amidinopenicillin, selective and highly effective against Gram-negative bacteria, especially *E. coli.* The side effects are few; most commonly reported are mild gastrointestinal symptoms (e.g. nausea) [6]. It has for a long time primarily been used for UTIs in the Scandinavian countries [7]. Several studies have shown pivmecillinam to be a safe and effective agent in the treatment of LUTIs [6, 8]. Even in countries where it is well used, there seems to be low rates of resistance [3, 8, 9] (e.g. 5% resistance in *E. coli* of the Danish primary sector [10]), and indications of a low rate of collateral damage and low risk of clonal spread of resistance [11].

The current international and European clinical practice guidelines for acute uncomplicated UTI recommend pivmecillinam as one of the first line drugs, the therapy is recommended to be 400 mg *b.i.d* for 5 days. Although, at the same time saying 400 mg *b.i.d* in any duration between 3 and 7 days is an appropriate choice, but pointing out a 5-day or a 7-day course probably is superior to a 3-day course [2]. The recommended duration is, however, based on evidence showing a 7-day course is better than a 3-day course [12]. Clinical studies on pivmecillinam treatment of LUTI caused by ESBL producing bacteria shows different success rates, i.e. 56% (200 mg *t.i.d*.) and 80% (mainly 400 mg *t.i.d*) [13, 14]. The higher success rate of 400 mg is supported by Monte Carlo simulations [15]. Thus, evidence point to a superiority of 400 mg dose.

With this knowledge, we wanted to compare the efficacy of a 400 mg *t.i.d.* 3-day versus 5-day regimen of pivmecillinam against uncomplicated LUTI.

## Methods

### Design

This study will be a prospectively, double-blinded, randomized, multi-center placebo-control trial, comparing the efficacy of a 3- and 5-day regimen of pivmecillinam 400 mg. *t.i.d.* against uncomplicated urinary tract infections. The study will be conducted according to good clinical practice (GCP) and each patient has to sign a consent form to be able to participate.

### Study population

#### Inclusion criteria

Women in the age of 18–70 years old, with clinical features of community acquired uncomplicated LUTI, with the ability to understand the study and give consent, can participate. Patients need to have a symptom cumulative score of ≥2 p (i.e. none, mild, moderate or severe, scoring 0–3, respectively) in accordance to the validated questionnaires for uncomplicated UTI [16]. The following symptoms are included:

- Dysuria (i.e. pain or burning sensation when urinating)
- Urgency (i.e. feeling an increased and more frequent need to urinate)
- Pollakisuria/frequency (i.e. urinating more frequently than usually)

An accepted definition of uncomplicated LUTI is symptoms in concordance with uncomplicated LUTI and significant bacteriuria of uropathogenic bacteria [1], but we choose not to include the latter criteria in our intention to treat (ITT) analysis. This is because we want the included patients in the ITT group to mimic the realistic settings as much as possible (i.e. in which patients with uncomplicated LUTI will receive antibiotic therapy without urine samples in most primary care settings).

#### Key exclusion criteria

- Allergy to penicillins and cephalosporines.
- Clear signs of (i.e. high fever ≥38,5 °C or flank pain/tenderness) or high suspicion of pyelonephritis.
- Any condition that may lead or predispose to complicated urinary infection (i.e. indwelling urinary catheter, pregnancy, immunosuppressive therapy, abnormal urinary tracts, recurrent UTI, serious neurological disease affecting the bladder).
- Porphyria or systemic primary carnitine deficiency or of the type organic aciduria (i.e. methylmalonic aciduria and propionacid anemia).
- Symptoms correlating with differential diagnosis (i.e. vaginal discharge or pain).
- Ongoing/current antibiotic therapy.
- Oesophageal stricture.
- Current intake of allopurinol (i.e. increases the risk of allergic skin reaction to mecillinam) or probenecid (i.e. decrease the renal excretion of mecillinam) or valproate.
- Currently part of another clinical trial. Inability/unable to understand and/or take part in the clinical trial

#### Settings and recruitment

The patients will be recruited in general practice (GP) clinics by GPs in Denmark. With nine collaborating clinics we aim to recruit 161 evaluable patients in each arm over app. 24 months. Female patients will, during this period, be screened for uncomplicated LUTI and recruited to the study. To receive the treatment goal as soon as possible the investigators will contact and support the GPs from time to time during the trial period.

#### Intervention

All patients included will receive pivmecillinam therapy for 3 days or 5 days. The 3-day regimen group will receive blinded placebo tablets on day 4 and 5. If a secondary antibiotic treatment is required due to treatment failure (i.e. ongoing or worsening symptoms), a new antibiotic cure will be chosen in line with the results from the pre-treatment (inclusion day) urine culture. The GPs will be at any time welcome to call the Department of Clinical Microbiology (DCM) (i.e. central investigation site) for consultation in the matter.

##### Drug processes and randomization

All drug process will be conducted according to the requirements of Good Manufacturing Practice (GMP) and Good Distribution Practice (GDP), in accordance with the Danish Health and Medicine Authority. The placebo tablets will be indistinguishable from the pivmecillinam tablets (i.e. in color, taste and design), and packaged in an identical blister-packaged (i.e. the commercial pivmecillinam blister-package). To guarantee blinding of both the patients, treating physicians and the investigators, a central randomization will be conducted by an independent qualified researcher. The randomization will be conducted in R 3.2.2 [17], with each kit given a number from 1 to 500 and randomized into 25 blocks including 20 kits each. The kits will be delivered to the collaborating clinics. Encrypted data will be stored centrally at the hospital research department and the collaborating hospital pharmacy for possible de-blinding.

#### Questionnaires

The GP will receive an inclusion-questionnaire that he/she will fill out on day 0 (inclusion day). The questionnaires his will include age, positive inclusion criteria (i.e. LUTI symptom load), exclusion criteria, debut date of symptoms, and annual incidence of LUTI. All participants will be given a diary-questionnaire for day 0 to day 7*.* In which they daily will answer questions on LUTI symptoms, score them of severity on daily bases and report on which day they feel completely cured (i.e. back to habitual conditions). They will also be asked to register impairment in daily life activities and quality (i.e. work, physical activity, sleep, mental well-being) similar to the symptom score, previous UTI, travel, weight (i.e. >70 kg) possible adverse effects, adherence to the therapy, use of analgesics and other optional information. The patient will also receive a second follow-up questionnaire which need to be filled out once anytime around day-28 (i.e. possible symptoms or side effects since end of therapy, current status of questions asked in the symptom-diary). All questionnaires on symptoms are constructed according to the validated questionnaires for LUTI symptom score [16]. They will be submitted together with the urine samples. All of the patients’ questionnaires will be so patients can easily understand and answer the questions.

#### Microbiological diagnostics

One pre-treatment urine sample (day 0) with optional urine-dipstick, and two control urine samples (day 8–10 and day 28–30, respectively) will be sampled from the patient. The three urine samples will be sent to the DCM, Hvidovre University Hospital, Denmark, for examination of the presence and count of uropathogenic bacteria. In presence of significant bacteriuria (i.e. ≥10^3^ bacteria/ml [*E. coli*] / ≥10^4^ bacteria/ml [other uropathogenic bacteria]) the isolates will also be examined for resistance mechanisms and patterns, minimal inhibitory concentration (MIC) to mecillinam (i.e. by E test, AB Biodisk, bioMe’rieux, Marcy-l’E’ toile, France) and determining the bacterial specimen (i.e. MALDI-TOF, Brukner, Germany). All urine samples will processed according to laboratory routine and susceptibility tested according to EUCAST guidelines [18]. During the study all significant uropathogenic isolates will be stored at minus 80 °C. At the end of the study these isolates will be genetically examined to determine their more specific identity/type, i.e. so we can evaluate if the potential relapse is either a true relapse or a reinfection (i.e. the same or different type of bacterial specimen).

## Data collection and procedures

### Standardization

To the most possible standardized and similar inclusion process all GPs participating in the study will be educated in the inclusion procedures by the principal investigator and one senior investigator whom will visit all clinics before the initiation of the study.

### Day 0/inclusion

When consulting a patient examined and considered a suitable participant according to the inclusion and exclusion criteria, the GP will give the patient the opportunity to participate in the study. If the patient is still willing to participate, the patient will sign a form of consent with the GP, be scored by the GP on initial symptom load, and give a urine sample which will be sent with the questionnaire to the central laboratory and optionally examined with dipstick

### Follow-up

The investigators at the central laboratory will be calling or sending text messages (patient’s decision) to the included patients to follow-up on the questionnaires and control urine-samples, as well as answering any questions the patients might have. The phone calls or text-messages are app. made on day 3–5, day 9–11 and day 28–30, respectively. However, not at any time will we try to increase the adherence to the treatment, since the study ought to be mirroring a realistic patient situation.

### Criteria for termination from the study

If a patient during the study would develop signs of upper UTI (i.e. pyelonephritis), treatment failure, serious adverse effects or allergic reactions to the medicine, or decision of withdrawal, she will have to terminate from the study and possibly get a new evaluation by a physician.

### Second medical consultation

Patients will be told at recruitment to visit the GP again if she develops worsening of symptoms or not experiencing any symptom relief during the study period. In that case the GP is able to choose a new treatment strategy for the patient and will have the potential microbiological diagnostic answers (i.e. from pre-treatment urine sample) to take in consideration in this approach.

## Outcome

### Primary endpoint

We have two co-primary endpoints:

- **Co-primary endpoint no. 1** –clinical efficacy (i.e. which group will be clinically cured fastest and proportion of patients cured at day four).
- **Co-primary endpoint no. 2** – bacteriological efficacy (i.e. proportion of patients bacteriologically cured at first control urine sample).

### Secondary endpoints

- Clinical signs and symptoms in uncomplicated LUTIs in women.
- Frequency and timing of relapse of symptoms and/or bacteriuria.
- Bacteriological findings (i.e. ESBL producing bacteria).
- Complications (i.e. urosepsis and/or pyelonephritis) within 28 days.

### Definitions

- Clinically cured is defined as cumulative symptom score of <2.
- Clinical relapse is defined as initial clinically cured followed by cumulative clinical score of ≥2 at day 28.
- Bacteriologically cured is defined as significant reduction (i.e. ≥ 10^2^ fold decrease in uropathogenic bacteria) or potentially full eradication of uropathogenic bacteria (i.e. sterile culture or asymptomatic contaminations of non-uropathogenic bacteria).
- Bacteriological relapse is defined as initially bacteriologically cured followed by significant growth at day-28, with the same bacteria. Growth of other uropathogenic bacteria is defined as reinfection.

### Procedures to evaluate endpoints

By comparing the questionnaires we can evaluate the clinical endpoints. By examining the urine samples we can evaluate the bacteriological endpoints. By asking for a questionnaire on day 28 we can evaluate any relapse/reinfection, hospitalization or secondary consultation, and afterwards look in the collaborating department of clinical microbiology’s databases for urosepsis or pyelonephritis.

## Power, sample size and analysis

### Definitions

The intention-to-treat (ITT) analysis will be consisting of all included patients (i.e. patients that present with symptoms of uncomplicated LUTI and are included in the study). The per-protocol (PP) analysis will be consisting of all the patients that follow-up with clinical and bacteriological data (i.e. excluding the dropouts). The need-to-treat (NTT) analysis will be stratified from the PP analysis to only include patient with true uncomplicated LUTI (i.e. symptoms and significant bacteriuria, only isolates susceptible to mecillinam).

### Power analyze

We hypothesize that there is a 15% absolute differences in efficacy between the groups, measured in clinical efficacy. We believe there to be an even bigger difference in bacteriological efficacy. Assuming an efficacy of 90 and 75% for 5 days and 3 days therapy, respectively, using a double-sided test with *p* < 0.05, and a statistical power of 0.8, we will need 113 patients in each group for the NTT analysis. With an estimated fall-out rate of app. 30% between each arm; 161 patients per group will need to be included for the PP analysis, and 230 in each group for the ITT analysis. There is a possibility for an interim analysis after 300 included participants, since we cannot reject a possibility for a higher (i.e. 20%) absolute efficacy.

## Data processing and statistical analysis

The source data is the GP’s and patient’s questionnaires and the diagnostics answers from the microbiological lab at the DCM. All source data will all be stored by the investigator and sponsor, in originals and scanned copies, at the DCM. The collected source data are transferred, logged and processed in the computers of the DCM. Analysis will be made in ITT, PP, and NTT form, respectively. All data will be analyzed coded, and only de-coded after the analysis is done and the analytic results are written in the relevant sections in the future article. The statistical software packages SAS (SAS Institute, Cary, NC) will be used for all analyses. Categorical data will be analyzed using a Fisher’s Exact test.

## Presentation of the results

Results (i.e. positive, negative and/or inconclusive) will be presented at scientific meetings, and in scientific publications in accordance to CONSORT 2010 statement [19].

## Ethical considerations

The study will comply with all the requirements to secure patients safety and to be carried out according to the Declaration of Helsinki, national laws and regulations. Ethical approval has been obtained by the Danish committee on biomedical research ethics for the capitol region of Denmark (No. H-4-2014-072). Approvals have also been obtained from the Danish National Health and Medicines Authority and the Danish Data Protection Agency. The study will be conducted according to the principle of Good Clinical Practice (GCP) and monitored by the GCP Unit of Bispebjerg Copenhagen University Hospital, Denmark. All patients will be included in concordance with rights of a research subject in clinical research, according to the national committee of health and research ethics document, which each subject will be provided the option to read before deciding to participate. The informed consent is obtained at the GP office, where the participant also will be informed about potential risks, benefits and procedures etc. based on an information sheet that the central investigators have prepared in concordance with above mentioned committees’ standards. The patient can withdraw their consent at any time without disadvantages.

### Data storage

The study will comply with the Danish regulation about management of personal information by the act on processing of personal data (Act No. 429 of 31 May 2000). All patient data will be carefully processed and stored at the Department of Clinical Microbiology, Hvidovre University Hospital, Denmark. All data will be anonymous in any possible future publication.

#### In trial intervention

The only time during the study in which we will intervene with antimicrobial therapy is if we during microbiological diagnostics find out that the patient is receiving a possibly ineffective therapy. This will for example be the case if we find causing uropathogenic bacteria to be:

1. resistant to mecillinam (i.e. a small proportion of uropathogenic bacteria) [9]
  and/or
2. a specimen to which pivmecillinam is not recommended for various reasons (non-susceptible, natural resistance etc.) - i.e. *Pseudomonas spp., Acinetobacter spp*., or *Enterococcus spp*. and other Gram positive uropathogenic bacteria etc.

We will then make contact and recommend another more suitable antimicrobial therapy.

### Adverse effects/risks

Pivmecillinam has been proven in RCTs to be safe and effective in the treatment of UTIs [6, 8, 20]. The risk off selecting and spreading off for mecillinam resistant bacteria is at very low risk [11], and mecillinam has a minor impact on the normal microflora [21]. Side effects are not more common than in any other penicillin, severe side effects are very rare, and the most common side effects are gastrointestinal complaints (i.e. nausea). Other common side-effects are rash and vulvovaginitis [6]. In the questionnaires the patients will be asked for possible adverse effects on day 1–7 and day 28. Any adverse reactions, serious adverse reactions or events during the study period will be reported according to GCP principles and in concordance with the Danish National Health and Medicine Authority.

## Discussion

Pivmecillinam is an excellent choice against urinary tract infections, and has shown to be both safe and effective against UTI [6, 12, 22], with a clear superiority to placebo [12] and superiority to sulftamethiazole [22]. In these days, with the concerning rise of multidrug-resistant bacteria, pivmecillinam has shown to be of an especial advantage; demonstrating low collateral damage (i.e. minor impact on the normal microflora [21], low resistance [3, 9], low selectivity and clonal spread of resistance [11]), as well as indication of clinical and bacteriological effective against ESBL producing Enterobacteriaceae [13, 23]. Hence, there are good evidences backing pivmecillinam as a first line of drug in the treatment of LUTI, as stated in the international guidelines [2], and as long time practiced in the Scandinavian countries.

Ferry et al. [12] have demonstrated that a 7-day regimen is superior to a 3-day regimen against uncomplicated LUTI. However, the current guidelines do not recommend a 7-day therapy, because of the increased risk of side effects (i.e. GI-problems). Nevertheless, there are no data comparing the 3-day vs. the 5-day therapy. With the 5-day therapy possibly to be more effective and with non-significant increased risk of side effects than the 3-day; a 5-day therapy might be a more cost-beneficial duration for the patient in the treatment of uncomplicated LUTI. Thus, with this study we want to strengthen and fill the gaps of evidence in our guidelines.

We have chosen the 400 mg dose *t.i.d.,* since that is the dose we recommend in Denmark; and we believe 200 mg *t.i.d* to be an insufficient dose (i.e. supported by pharmacokinetic mouse models [15] and the recent study from Soraas et al*.* [14]). With this knowledge, we believe it to be unethical of us to use a 200 mg dose, and have therefore excluded a treatment arm with 200 mg.

### Design and definitions

We designed this protocol similarly to previous similar clinical studies on uncomplicated LUTI [12, 22, 24, 25], in order to be able to compare results in the future in a more accurate way. We define uncomplicated LUTI in concordance with the acknowledged definition [1, 26], with one difference; we choose to include post-menopausal women in our study, since we do not necessarily believe them to always be of complicated LUTI. Hence, if a post-menopausal woman has no other complicated conditions (see exclusion criteria) we believe her to be a potential candidate.

### Procedures

We want the inclusion procedures to be as realistic as possible. Therefore, the consulting with the GPs will be changed as little as possible and restricted to the inclusion day. However, patients will be clearly instructed to return to the GP as usual at sign of treatment failure, or if they wish to consult for any other reason.

### Outcomes

The primary outcome we want to focus on is the clinical effect, since that is of most importance to the patient. However, it is important to know that the bacteria disappear and not cause clinical relapses in the future; therefore, we included two follow-up urine sample at day-4 and day-28, respectively.

#### Scientific and societal perspective

This study will give us the knowledge of what drug therapy of pivmecillinam that is most effective and sufficient. If the 5-day therapy will show to be significantly superior to the 3-day regimen, the current guidelines will have to be changed in best interests of the patients. Also through the perspective of the patients, it will be beneficial if the 3-day regimen is proven to be non-inferior to the 5-day regimen (i.e. higher rate of compliance, and less antimicrobial consumption cause less economic and ecological loads), and we can keep using the 3-day regimen in concordance with the principles of evidence-based medicine.

## Conclusion

We believe this study will fill the scientific gaps and provide sufficient evidence for the most efficient dose duration of pivmecillinam against uncomplicated lower urinary tract infections. Thus, strengthening the guidelines for one of the most common community acquired infections in our society.

## Abbreviations

b.i.d: Two times daily
DCM: Department of clinical microbiology
ESBL: Extended Spectrum Beta-Lactamase
GCP: Good clinical practice
GP: General practitioner
ITT: Intention-to-treat
LUTI: Uncomplicated Lower Urinary Tract Infections
NTT: Need-to-treat
PP: Per-protocol
t.i.d.: Three times daily
